# CoFe_2_O_4_-Quantum Dots for Synergistic Photothermal/Photodynamic Therapy of Non-small-Cell Lung Cancer Via Triggering Apoptosis by Regulating PI3K/AKT Pathway

**DOI:** 10.1186/s11671-021-03580-5

**Published:** 2021-07-28

**Authors:** Jingfeng Liu, Xiaoying Shi, Rongjun Zhang, Miaomiao Zhang, Juan He, Jian Chen, Zheng Wang, Qingwen Wang

**Affiliations:** 1 Department of Rheumatism and Immunology, Peking University Shenzhen Hospital, Shenzhen Peking University-The Hong Kong University of Science and Technology Medical Center, Shenzhen, 518036 Guangdong China; 2 Shenzhen Key Laboratory of Immunity and Inflammatory Diseases, Peking University Shenzhen Hospital, Shenzhen, 518036 Guangdong China; 3 Cardiovascular Hospital, No. 1 Hospital of Xi’an City, Xi’an, 710002 China; 4 Cancer Research Institute, School of Basic Medical Sciences, Southern Medical University, Guangzhou, 510515 Guangdong China; 5 School of Advanced Materials and Nanotechnology, Xidian University, Xi’an, 710126 China

**Keywords:** Quantum Dots, NSCLC, Photodymatic therapy, ROS, PI3K/Akt

## Abstract

Non-small-cell lung cancer (NSCLC) has become the second most diagnosed malignant tumors worldwide. As our long-term interests in seeking nanomaterials to develop strategies of cancer therapies, we herein constructed novel CoFe_2_O_4_-quantum dots (QDs) with outstanding synergistic photothermal/photodynamic property which suppressed NSCLC efficiently without apparent toxicity. We showed that the combination of CoFe_2_O_4_-QDs + NIR treatment induces apoptosis of NSCLC cells. In addition, the CoFe_2_O_4_-QDs + NIR treatment also promotes reactive oxygen species generation to trigger cell death through regulating PI3K/AKT pathway. Moreover, the CoFe_2_O_4_-QDs + NIR treatment successfully eliminates tumor xenografts in vivo without apparent toxic effects. Taken together, we reported that the novel nanomaterials CoFe_2_O_4_-QDs could exhibit enhanced synergistic photothermal therapy and photodynamic therapy effect on killing NSCLC without toxicity, which could be a promising photosensitizer for NSCLC therapy.

**Supplementary Information:**

The online version contains supplementary material available at 10.1186/s11671-021-03580-5.

## Introduction

Cancer is the leading cause of death and brings huge burden for the family and society, among which lung cancer ranks the second most diagnosed cancer and the first of cancer-related death in 2020 [1, 2]. As reported, non-small-cell lung cancer (NSCLC), which accounts about 85% of all lung cancers, is characterized in high incidence and mortality [3, 4]. Recently, in spite of surgical options, great effort has been given to develop chemotherapies or immunotherapies to treat NSCLC. For example, EGFR-mutant inhibitors and KRAS inhibitors have been proved effective and there still more novel ALK inhibitors are ongoing [5–9]. Anti-PDL1 and anti-CLTA4, such immune check point inhibitors also bring promising efficacy and prolong survival benefit [10–12]. However, the responsive rate toward these drugs differs from patient to patient and the side effects, especially drug resistance, should not be neglected [13, 14]. Therefore, to develop novel therapeutic strategies, which are less invasive, is an urgent and also a necessity for NSCLC research and clinical treatments.

Based on recent progress, using nanomaterials to perform photothermal therapy (PTT) and photodynamic therapy (PDT) has aroused tremendous attention and reached great development as an anti-cancer strategy and may be an alternative option in clinical treatment [15–18]. Nanomaterial-based PTT and PDT is characterized in less invasion and low toxicity, which with little chance to induce drug resistance [19–23]. With the collaboration of light, mostly NIR, localized nanomaterials can raise the temperature within the tumor and convert oxygen to cytotoxicity reactive oxygen species (ROS), which causes cell death in order to eliminate tumors [24]. In this context, the nanomaterial plays a key role in here to influence the efficacy and guarantee the safety. Although such nanomaterials have included metal nanostructures [25], carbon-based materials [26, 27], polymeric nanoparticles (PNPs) [28] or semiconductor compounds [29], they have their own limitations. For example, carbon-based materials are costly and have unsatisfactory suspension property, which limits its application in large scale and clinical potentiality. Therefore, more attempts should be addressed to generate more suitable nanomaterials for further usage.

Recent years, quantum dots (QDs), as novel nanomaterials, have received great attraction in biomedical applications because of their good bio-compatibility, solubility and the most important their superior photo-stability and facile surface functionalization property [30–33]. Taking advantage of these properties, several reports have used QDs as novel PDT reagents and can be designed to be accompanied with other biomolecules to enhance the efficacy of PDT in cancer treatment. For example, Meng and colleagues reported a multi-functional GQD@MnO_2_ induced by two-photo excitation to improve the PDT efficacy [34]. In addition, Kuo and colleagues generated nitrogen-doped QDs by functionalized them with amino molecules, which enhanced the PDT efficiency as well [35]. Inspired by these interesting findings, we sought to develop novel QDs combined with non-noble metal-based nanomaterial which may bring PTT and PDT synergistic effects in one nano-system. For example, Co-based nanomaterial is well-studied non-noble metal-based nanomaterial, which is known for used as PTT agents for tumor therapy or imaging [36]. Therefore, we suggested that designing Co-based QDs may bring enhanced PTT/PDT synergistic effects.

In this study, we synthesized novel nanomaterials CoFe_2_O_4_-QDs which exhibits enhanced PTT and PDT synergistic effects on killing NSCLC without toxic effects in vitro and in vivo, which could be a promising photosensitizer for NSCLC therapy.

## Material and Methods

### Synthesis of CoFe_2_O_4_-QDs

The CoFe_2_O_4_-QDs were synthesized though hydrothermal method. Typically, 0.238 g CoCl_2_·6H_2_O and 0.808 g Fe(NO_3_)_3_·9H_2_O were dissolved in 10 mL H_2_O and 10 mL propylene glycol mixture solvent, and then stirred for 10 min. Then 4 mL diethanol amine was added into the solution drop by drop, followed by stirring for 30 min. Then the obtained slurry was transformed to a 50 mL stainless Teflon-lined autoclave. The autoclave was maintained at 160 ℃ for 3 h in an oven. The CoFe_2_O_4_-QDs were collected by centrifuging at 8500 rpm for 10 min and then rinsed by deionized water and ethanol successively. Reagents and materials used in this study could be found in Table 1.

**Table1:** Reagents or resource used in this study

Reagents or resource	Source	Identifier
CoCl_2_·6H_2_O	Sigma-Aldrich	Cat# 255599
Fe(NO_3_)_3_·9H_2_O	Sigma-Aldrich	Cat# 216828
Propylene glycol	Sigma-Aldrich	Cat# 398039
RPMI-1640	Gibco	Cat# 11875119
Fetal bovine serum (FBS)	Gibco	Cat# 10270106
Penicillin–Streptomycin	Gibco	Cat# 15140122
Endothelial cell growth medium	Sigma-Aldrich	Cat# 211–500
CCK-8 Kit	Dojindo	Cat# CK04-05
Annexin-V/PI apoptosis kit	BD Bioscience	Cat# 556547
DCFH-DA	Abcam	Cat# ab113851
NAC inhibitor	Sigma-Aldrich	Cat# A7250
RIPA lysis buffer	Thermo Fisher	Cat# 89900
MatriGel	Corning	Cat# 354277

### Characterization of CoFe_2_O_4_-QDs

The morphology and size of prepared CoFe_2_O_4_-QDs were determined by TEM and EDS system. The crystal structure was analyzed by X-ray diffractometer (Bruker Germany) equipped with Cu Ka radiation (*k* = 0.15406 nm). The absorbance spectrum of CoFe_2_O_4_-QDs was detected by SHIMADZU UV-2600 spectrophotometer. The element valence states of CoFe_2_O_4_-QDs were determined by X-ray photoemission spectroscopy measurements (XPS, VG ESCALAB 220I-XL, USA). The thermal image was recorded with IR thermal camera (FLIR E50, USA).

### Cell Culture

NSCLC cell line NCI-H460 (H460) and A549 and Human umbilical vein endothelial cells (HUVECs) were obtained from ATCC and tested for micro-plasma negative. H460 and A549 cells were cultured in RPMI-1640 supplemented with 10% fetal bovine serum (FBS) and 1% Penicillin–Streptomycin (Gibco). HUVECs were cultured in endothelial cell growth medium (Sigma, #211-500). All the cells were kept in dark humidity 37 ℃ incubator with 5% CO_2_.

### Cytotoxicity Detection

Various working concentrations (0.1, 0.5, 1.0, 2.0 mg/mL) of CoFe_2_O_4_-QDs were added and cultured with HUVECs for 24 h. After incubation, culture medium was changed and CCK-8 regent was added to each well followed by 1 h incubation. Then, plates were measured at 450 nm with EnSpire™ Multimode Plate Reader. The ratio of cell viability was taken as 100% in control HUVECs.

### Apoptosis Analysis

H460 and A549 cells (2 × 10^5^) were cultured in 6-well plates overnight before treated with 1.0 mg/mL CoFe_2_O_4_-QDs combined with NIR laser of 808 nm for 5 min. Then cells were washed and stained with Annexin-V/PI apoptosis kit (BD; #556547) following manufacturer’s instructions. As for HUVECs apoptosis assay, HUVECs were incubated with different concentration of CoFe_2_O_4_-QDs. The apoptosis ratio was determined as described above.

### Cellular ROS Detection

H460 and A549 cells were cultured in 6-well plates overnight. Cells were incubated with or without 1.0 mg/mL for 1 h and treated with NIR laser of 808 nm for 5 min. After treatments, DCFH-DA was added and incubated for 30 min followed by FACS detection with excitation/emission at 485 nm/535 nm. As for ROS inhibition assay, ROS inhibitor NAC (Sigma; A7250) was added according to manufacturer’s instruction. Data were further quantified with Flow-jo software.

### Western Blot Analysis

H460 and A549 cells were treated as apoptosis assay, and the whole cell protein was extracted using RIPA lysis buffer. Western blot detection was carried out as described before [37]. The antibodies used in this study were listed below: rabbit polyclonal anti-Bcl-2 (abcam; ab59348), rabbit monoclonal anti-Bax (abcam; ab32503), rabbit polyclonal anti-*P*-PI3K (Bio-Vision; 3152-100), rabbit monoclonal anti-*P*-AKT-S473 (CST; 4060S), rabbit monoclonal anti-β-Actin (CST; 4970S), anti-rabbit IgG HRP-linked antibody (CST; 7074S). Quantification was determined by using Image-J software.

### In vivo Study of Anti-NSCLC Effect of the Combination of CoFe_2_O_4_ and NIR Treatment

To determine the tumor killing ability of CoFe_2_O_4_-QDs, H460 cells were subcutaneously implanted with 50% MatriGel into NSG mice (*N* = 8 each group). 4–6-week-old male M-NSG mice were obtained from Shanghai Model Organisms (#NM-NSG-001) for all the in vivo experiments. When the tumor was visualized and the volume reached nearly 5 mm × 5 mm, all the mice were randomly divided into four groups, named as Control, NIR only, CoFe_2_O_4_-QDs only and CoFe_2_O_4_-QDs + NIR group, respectively. Then mice in CoFe_2_O_4_-QDs only and CoFe_2_O_4_-QDs + NIR group were intratumorally injected with 50 μL of CoFe_2_O_4_ (5.0 mg/kg) based on our previous work [37], while Control and NIR group was injected with 50 μL of PBS. After injection, NIR 808 nm laser (1 W/cm^2^) was performed in NIR and CoFe_2_O_4_-QDs + NIR group for 10 min, which was monitored by infrared thermal imaging equipment. Tumor volume was recorded every day and calculated with the formula *V* = length × width^2^/2. Once the diameter of tumor xenografts in remaining mice reached almost 15 mm, mice were sacrificed and tumor xenografts were photographed and stored for further detection. All the animal experiments and protocols were approved by Institutional Animal Care and Use Committee (IACUC) and Animal Welfare Committee of Peking University Shenzhen Hospital.

### H&E and Immunohistopathology Staining Analysis

For pathological assessment, the tumor xenografts (*N* = 3) were harvested one day after treatment in each group and then fixed in 10% buffered formalin following embedded in paraffin for H&E staining and IHC detection. For in vivo toxicity evaluation, the kidney, liver, lung, heart and spleen of the mice were extracted and fixed for pathological assessment. For IHC staining, anti-Ki67 antibody (Abcam; ab15580) was used. Quantification of IHC positive area was conducted by software Fiji.

### Statistical Analysis

For all experiments, “*N*” represents the number of repeated times or the number of mice used as indicated in the figure legend. Student’s *t*-test or one-way ANOVA was used for statistical comparisons. *P* < 0.05 is considered statistically significant while “ns” displays non-significant. *P* < 0.05, *P* < 0.01 and *P* < 0.001 are indicated with “*”, “**” and “***” asterisks, respectively. Data were analyzed using GraphPad Prism 5.

## Results

### The Characteristics of Novel CoFe_2_O_4_-QDs

**Fig. 1:**
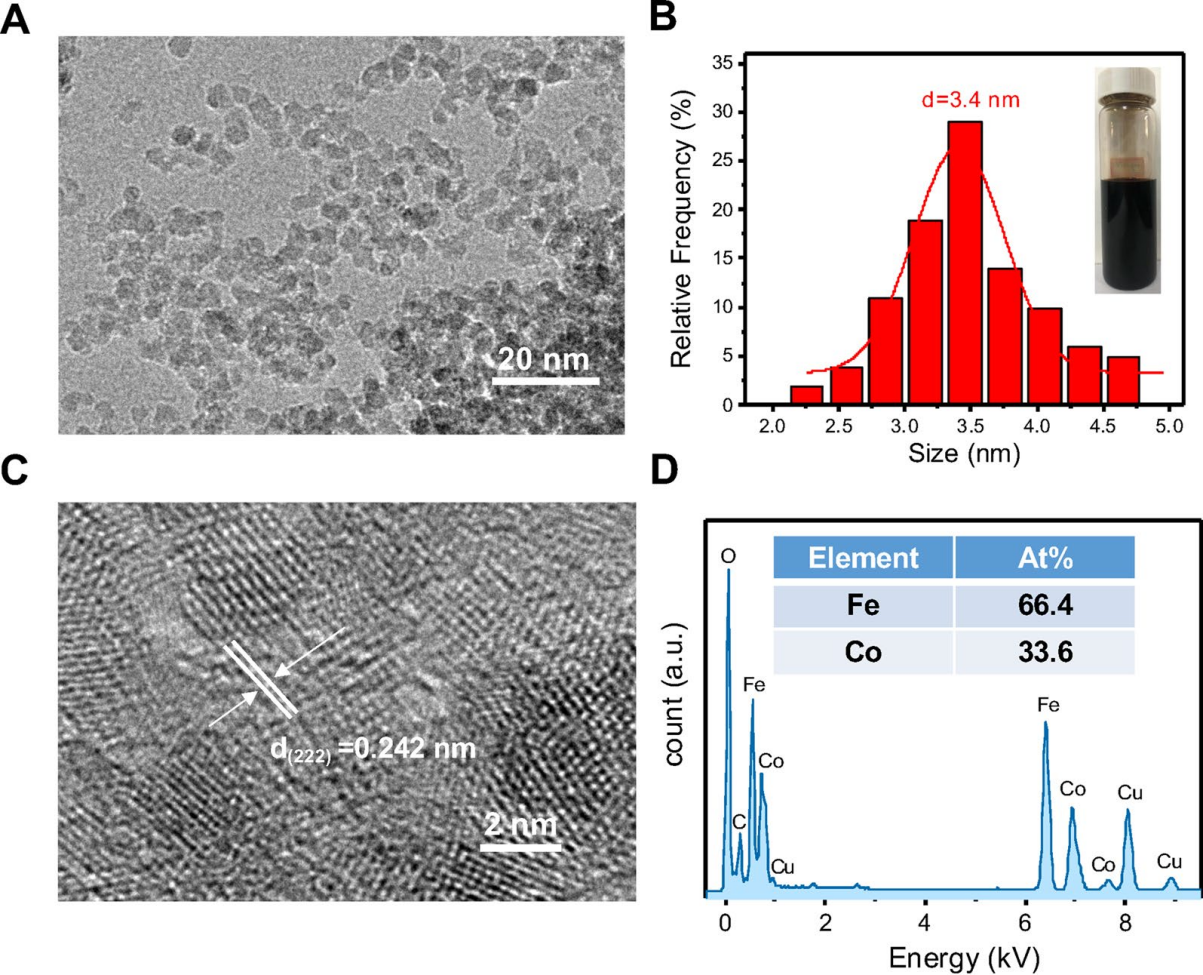
Preparation and characterization of CoFe_2_O_4_-QDs. **a** The representative TEM image of as-prepared CoFe_2_O_4_-QDs. Scale bar, 20 nm. **b** A global analysis of the size of CoFe_2_O_4_-QDs with the average diameter 3.4 nm. The inset is the representative digital photo of the CoFe_2_O_4_-QDs suspension. **c** The lattice fringes of as-prepared nanocrystals correspond to CoFe_2_O_4_-QDs HRTEM image. Scale bar, 2 nm. **d** The as-prepared CoFe_2_O_4_-QDs exhibited uniform distribution of Co, Fe and O. The representative elements map was shown

Firstly, we constructed the CoFe_2_O_4_-QDs using hydrothermal approach which is low cost and simple to perform. The TEM image of CoFe_2_O_4_-QDs was shown in Fig. 1a, presenting a uniform and stable pattern with the diameter around 3.4 nm (Fig. 1b). The as-prepared CoFe_2_O_4_-QDs were dark brown in color (Fig. 1b) and with excellent solubility in water. Furthermore, the high-resolution TEM image (Fig. 1c) displayed the lattice spacing of (222) is about 0.242 nm which is consistent with the crystal parameters of CoFe_2_O_4_-QDs [38, 39]. In addition, the element spectrum (Fig. 1d) further confirmed the element component of the CoFe_2_O_4_-QDs is Co and Fe, and the atom ratio of Co and Fe was about 1:2. These data displayed a successful construction of CoFe_2_O_4_-QDs for our further research.

### The Physical Property Detection of CoFe_2_O_4_-QDs

**Fig. 2:**
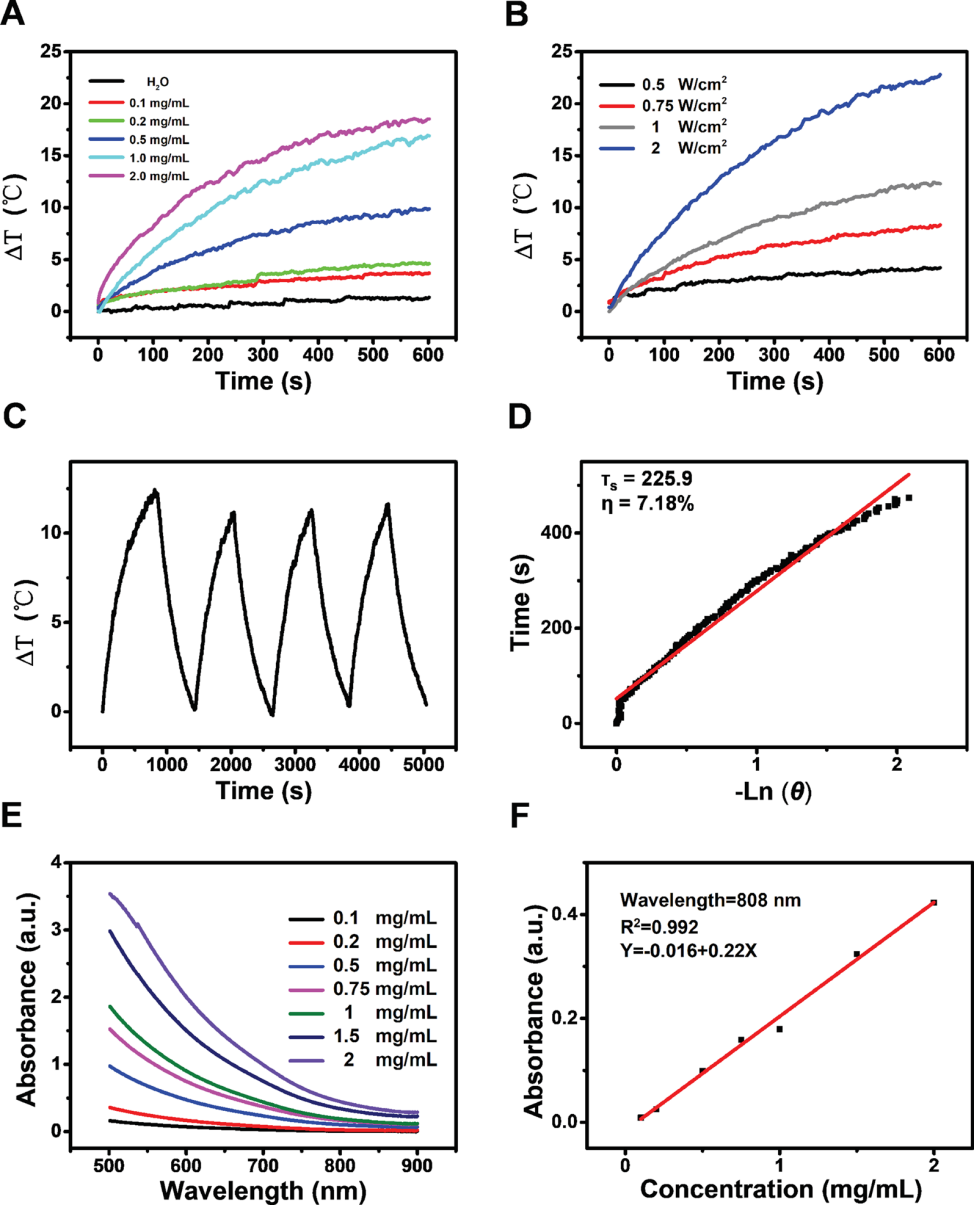
The property evaluation of CoFe_2_O_4_-QDs. **a** The photothermal conversion of CoFe_2_O_4_-QDs was determined under different concentrations. The heating curves are shown. **b** The irradiation energy dependent manner of CoFe_2_O_4_-QDs is shown at different power densities (0.5–2.0 W/cm^2^). **c** CoFe_2_O_4_-QDs have stable photothermal conversion detected with 4 cycles of heating-cooling continuous irradiation (1.0 W/cm^2^). **d** The efficiency of CoFe_2_O_4_-QDs photothermal conversion. **e**, **f** The relationship of wavelength and absorbance of CoFe_2_O_4_-QDs

In order to determine the physical properties of prepared CoFe_2_O_4_-QDs, we performed several detections after construction. With the NIR absorbance determination test, CoFe_2_O_4_-QDs showed proper photothermal conversion in a concentration dependent manner and the temperature increments (Δ*T*) could be adjusted from 0.3 to 18.9 °C (Fig. 2a). In addition, at the concentration 1.0 mg/mL of CoFe_2_O_4_-QDs, by increasing the NIR radiation power from 0.5 to 2.0 W/cm^2^, the ΔT could be tuned from 0.8 to 24.3 °C (Fig. 2b). These data suggested that the photothermal conversion performance of CoFe_2_O_4_-QDs was dependent on its concentration and the irradiation power. Furthermore, the stability of CoFe_2_O_4_-QDs triggered photothermal conversion was determined with period irradiation (Fig. 2c). Although the calculated light-to-heat conversion efficiency was 7.18% (Fig. 2d), it’s enough to be accompanied to enhance the PDT effect of CoFe_2_O_4_-QDs. Moreover, the longest wavelength of CoFe_2_O_4_-QDs can absorb light is about 808 nm (Fig. 2e, f). Taken together, these data suggested that CoFe_2_O_4_-QDs could be developed into a promising PTT/PDT synergistic agent for alternative tumor killing therapy.

### Cytotoxicity Assessment of CoFe_2_O_4_-QDs Toward Normal Cells

**Fig. 3:**
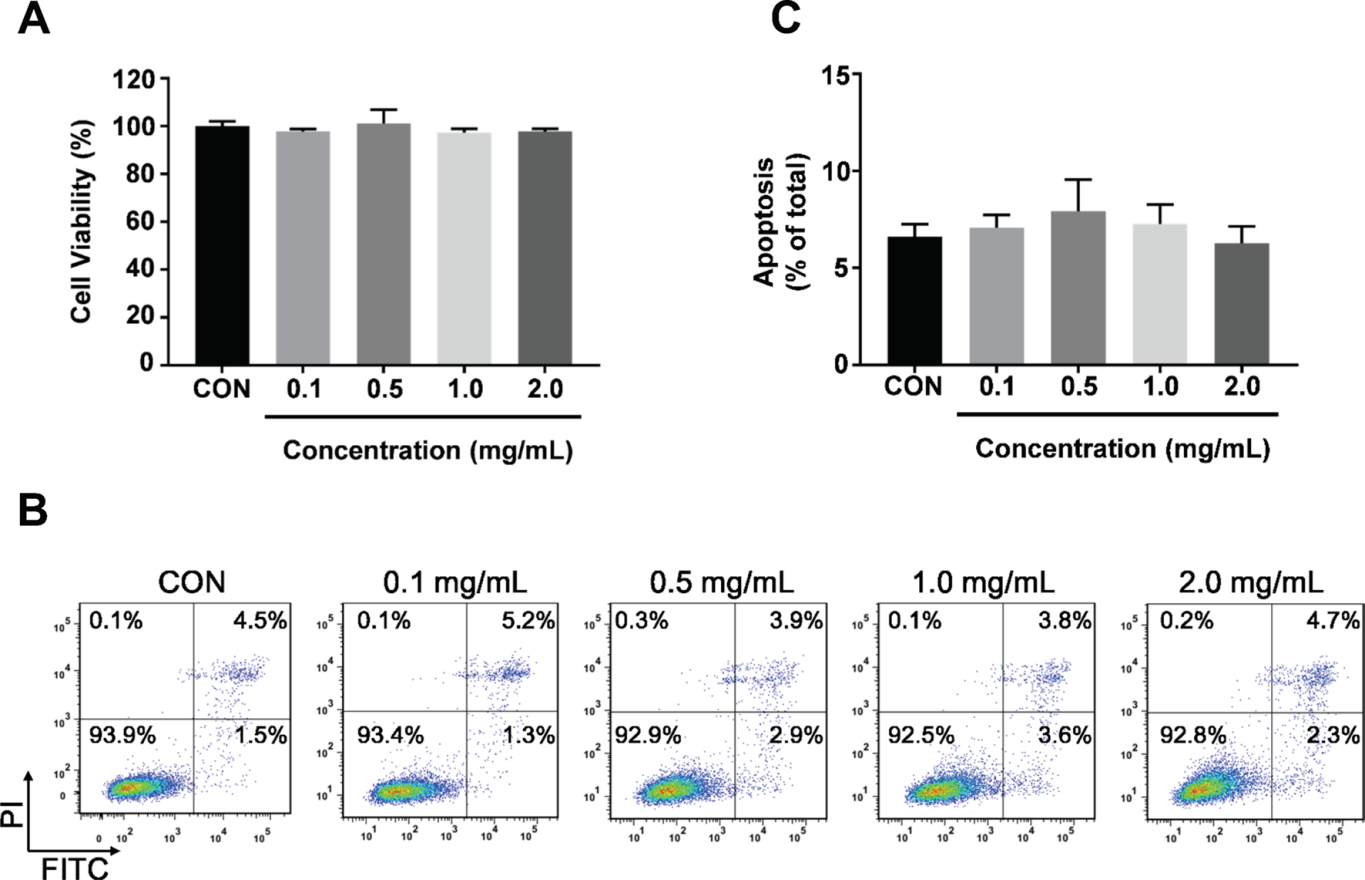
In vitro assessment of cytotoxicity of CoFe_2_O_4_-QDs toward normal cells. **a** CCK-8 cell viability assay in HUVECs was performed under different concentrations of CoFe_2_O_4_-QDs. The viability of control group was taken as 100%. The data were shown as mean ± SD, *N* = 3. **b** HUVECs apoptosis was determined by FACS detection. Representative images of indicated concentrations are shown, *N* = 3. **c** The quantification of apoptosis ratio. They show non-significance comparing to control group. The data were shown as mean ± SD

Since nanoparticles are widely used as drug delivers or intra-medium for tumor therapies, the cytotoxicity of CoFe_2_O_4_-QDs toward normal cells especially human vascular epithelial cells should be confirmed for further usage. Therefore, from the previous results, we tested different concentrations (0.1, 0.5, 1.0 and 2.0 mg/mL) of CoFe_2_O_4_-QDs. After co-cultured with HUVECs (normal human epithelial cell line), CCK-8 reagent was added for the detection of cell viability. There was no obvious cytotoxicity observed comparing to control group (Fig. 3a). In this context, further apoptosis assay was performed to achieve consistent results with the same conditions (Fig. 3b). The quantification of apoptosis rate indicated no significant difference comparing to control group (Fig. 3c). These data showed that CoFe_2_O_4_-QDs had no apparent toxic effect on normal cells, which indicated that CoFe_2_O_4_-QDs had the potential to be used as intermedium for drug deliver.

### Combination of NIR and CoFe_2_O_4_-QDs Induces Apoptosis of NSCLC

**Fig. 4:**
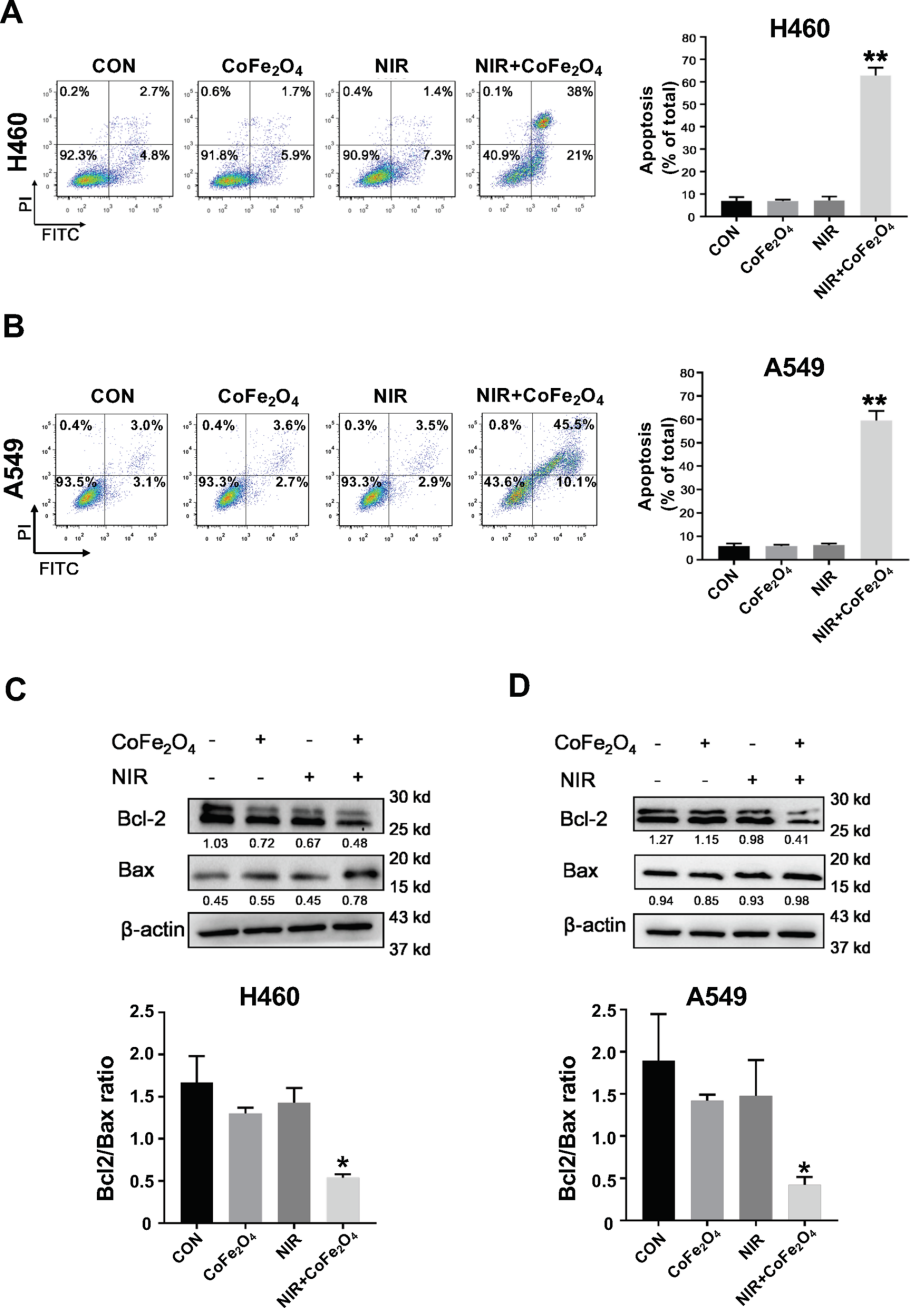
In vitro combination of CoFe_2_O_4_-QDs and NIR induces NSCLC apoptosis. **a**, **b** H460 and A549 NSCLCs were treated with the combination of CoFe_2_O_4_-QDs and NIR laser for 5 min. The apoptotic cells were evaluated by Annexin-V staining and detected by FACS. Quantification is shown respectively as well. The data were shown as mean ± SD, *N* = 3. **P < 0.01 vs Control, NIR, CoFe_2_O_4_ groups. **c**, **d** The protein level of Bcl-2 and Bax were determined by Western blot analysis in NCI-H460 and A549 NSCLC after treatment. Representative images are shown. The quantification is shown after calibrating to the expression of internal control β-Actin. The data were shown as mean ± SD, *N* = 3. *P < 0.05 vs Control, NIR, CoFe_2_O_4_ groups

To determine the potential NSCLC cancer killing ability of CoFe_2_O_4_-QDs, NIR laser (808 nm) irradiation was performed combined with incubation of CoFe_2_O_4_-QDs in vitro. Then, apoptosis assay was carried out after treatments, both H460 and A549 cells revealed aggressive apoptosis rate with the combination of CoFe_2_O_4_-QDs and NIR laser (Fig. 4a, b). Quantification showed significant difference comparing to control group, while CoFe_2_O_4_-QDs only or NIR only groups showed no difference which indicated that CoFe_2_O_4_-QDs plus NIR could induce anti-NSCLC effect (Fig. 4a, b). It is well known that the alteration of the protein level of Bcl-2/Bax is important to determine whether the cells would undergo apoptosis [40]. Consistent with this idea, the protein level of Bcl-2 and Bax was determined in both H460 and A549 cells after treatments (Fig. 4c, d). As respected, the data also showed that the ratio of Bcl-2/Bax decreased, which was regarded as the marker of mitochondria-mediated apoptosis. Therefore, we presented that CoFe_2_O_4_-QDs plus NIR gives rise to anti-NSCLC effect through activating mitochondria-mediated apoptosis pathway.

### Combination of CoFe_2_O_4_-QDs and NIR Induces ROS Generation via PI3K/AKT Pathway

**Fig. 5:**
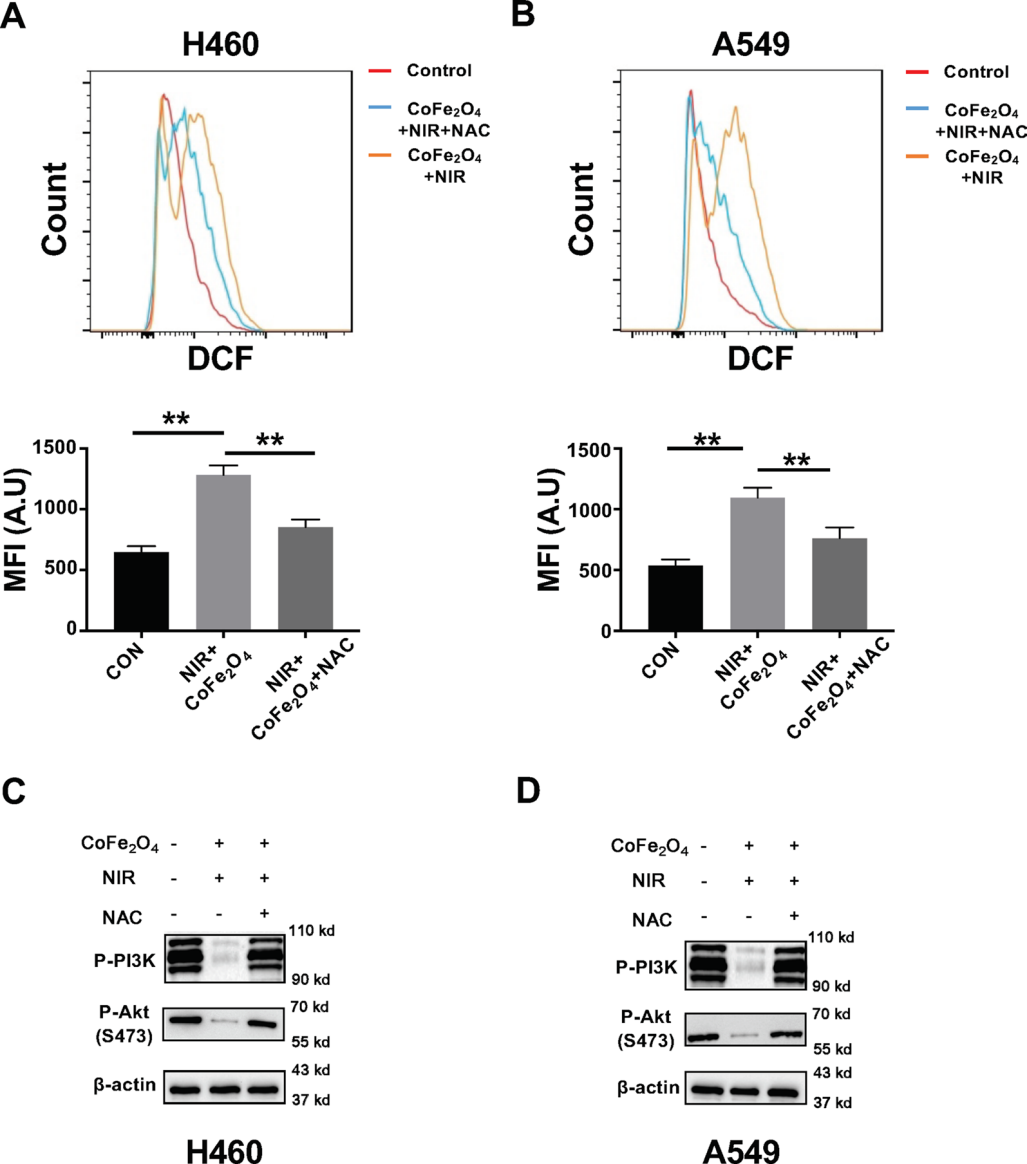
The combination of CoFe_2_O_4_-QDs and NIR induces ROS generation through regulating PI3K/AKT pathway. **a**, **b** NCI-H460 and A549 NSCLCs were treated with the CoFe_2_O_4_-QDs and NIR laser with or without NAC for 5 min. The ROS level was detected by FACS and the mean fluorescent intensity was quantified, respectively. The data were shown as mean ± SD, *N* = 3. **P < 0.01. **c**, **d** The protein level of *P*-PI3K and *P*-AKT was determined by Western blot analysis. Representative images are shown, *N* = 3

Mitochondria dysfunction always leads to upregulated level of ROS generation, which cause cell death in NSCLC. In this context, we performed ROS detection after CoFe_2_O_4_-QDs plus NIR treatment in H460 and A549 cells. The results showed that an immense releasing of ROS in combination group, which indicated enhanced PDT effect could be induced by CoFe_2_O_4_-QDs even with low photothermal transmission efficiency (Additional file 1: Fig. S1A, B). Furthermore, the related protein level of PI3K/AKT signaling pathway was also reduced, which suggested that the alteration of ROS was regulated by PI3K/AKT pathway, which leads to the alteration of Bcl-2/Bax protein expression level (Fig. 4c, d, Additional file 1: Fig. S1C, D). To confirm this idea, the ROS inhibitor NAC was added to reverse the phenomenon (Fig. 5a, b). Then, the expression of PI3K/AKT was determined to be rescued after NAC treatment, which further confirmed that the ROS releasing after CoFe_2_O_4_-QDs plus NIR treatment was regulated by PI3K/AKT pathway (Fig. 5c, d). These findings strongly support the idea that the combination of CoFe_2_O_4_-QDs and NIR can lead to synergistic PTT and PDT effect in killing NSCLC cells by inducing mitochondria dysfunction (ROS) dependent apoptosis.

### In vivo Anti-NSCLC Assessment of Combination of CoFe_2_O_4_-QDs and NIR

**Fig. 6:**
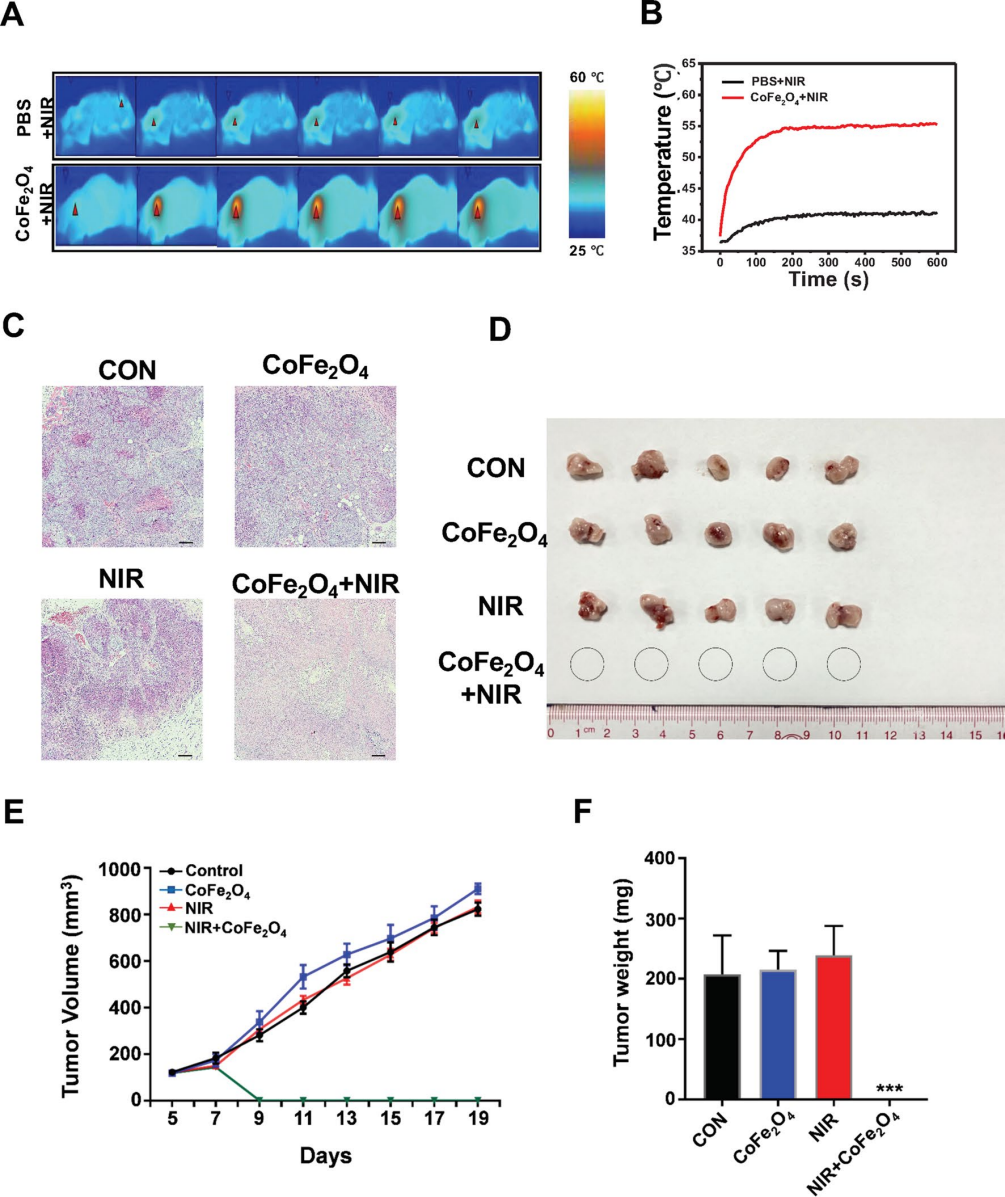
In vivo tumor killing assessment of the combination of CoFe_2_O_4_-QDs and NIR treatment. **a** The representative infrared thermal images of M-NSG mice bearing NCI-H460 tumor xenografts are shown. **b** The temperature curve shows the increasing temperature within tumor xenografts under NIR irradiation. **c** H&E pathological staining of each group was photographed 1 day after treatment. Apparent necrosis could be observed in combination group. Representative images are shown, *N* = 3. **d** The photograph of the xenografts in each group after the mice were sacrificed, *N* = 5. **e**, **f** The growth curve and weight of tumor xenografts in each group was recorded. The data were shown as mean ± SD, *N* = 5. ***P < 0.001

Based on the in vitro results, we next investigated the anti-NSCLC effect of CoFe_2_O_4_-QDs and NIR combination treatment on NSCLC tumor bearing mice model. M-NSG mice were subcutaneously implanted with H460 cells. After intratumorally injected with CoFe_2_O_4_-QDs, the NIR laser irradiation caused a rapid temperature raise to around 56 °C under the monitor of thermal detection equipment (Fig. 6a, b). Moreover, histopathological staining showed extensive necrosis area observed in combination group indicating that CoFe_2_O_4_-QDs plus NIR treatment caused tumor cell death as a result of tumor elimination (Fig. 6c, d). Further IHC staining also showed that Ki-67 positive area was aggressively shrunk comparing to other groups after the combinational treatment indicating the treated tumor xenografts could no longer proliferate (Additional file 2: Fig. S2A, B). Next, we followed up for 12 days after CoFe_2_O_4_-QDs and NIR treatment. As we expected, the size and weight of the tumor xenografts in other groups remarkedly grow but not in the CoFe_2_O_4_-QDs and NIR treatment group (Fig. 6e, f), supporting the idea that CoFe_2_O_4_-QDs and NIR combined treatment could completely eliminate the tumor xenografts in vivo. As for the cytotoxic effect of CoFe_2_O_4_-QDs, at least in our observation period, no obvious adverse effect was detected from the results of histopathology analysis within important organs of mice (Additional file 2: Fig. S2C). The above data provided strong evidence that CoFe_2_O_4_-QDs could be developed as a novel PTT/PDT reagent for NSCLC treatment.

## Discussion

In recent years, the research of developing anti-NSCLC strategies has achieved tremendous progresses. Both the precision medicine targeting specific mutant oncogene-addicted NSCLC and immune checkpoint blockade therapies bring promising future in clinical treatments [41, 42]. However, given the complexity and heterogenicity of tumor microenvironment and underlying risk of losing tumor antigen, it remains a bottleneck to lower the drug resistance rate following the immune evasive status, which leads to tumor relapse in a short time. Therefore, seeking novel treatments or intermedia for NSCLC therapies is an urgent. Among the emerging approaches, nanomaterials have been valued and listed front as effective cancer killing agents. Taking advantages of their small size, good biocompatibilities and thermal transmission abilities, several nanomaterials exert excellent cancer killing abilities in recent researches [43].

In our study, we developed a novel CoFe_2_O_4_-QDs which could be applied as an intermedia for NSCLC treatments via inducing tumor cells apoptosis with synergistic PTT and PDT effects. Like other nanomaterials, CoFe_2_O_4_-QDs exhibited excellent biocompatibilities in our studies which showed no obvious toxicity toward normal cells and major organs. Although we found the thermal transmission rate is not high as other nanomaterials, it’s enough for CoFe_2_O_4_-QDs to induce cancer cell apoptosis under the NIR laser activation. CoFe_2_O_4_-QDs shows good linear relationship with the light absorbance in this study and potentially generate ROS with the combination of NIR laser, which further prove that CoFe_2_O_4_-QDs can act as advantaged photosensitizer. We next can further optimize the structure or add thermal sensitive elements to CoFe_2_O_4_-QDs which could reach a higher thermal transmission rate for better synergistic PTT and PDT effects [44, 45]. Moreover, applying chemical drugs or antibodies on the surface of CoFe_2_O_4_-QDs is also feasible, which may bring superior killing efficiency. For example, the approach of linking anti-PDL1 or anti-CTLA4 antibodies to CoFe_2_O_4_-QDs could be a promising combinational therapy in breaking immune suppressive microenvironment within tumors which is our next interests to make a full usage with CoFe_2_O_4_-QDs.

Besides, the mechanism of CoFe_2_O_4_-QDs in killing NSCLC was also elucidated in this study. We confirmed that CoFe_2_O_4_-QDs induced NSCLC apoptosis mainly through ROS secretion after NIR laser activated synergistic PDT and PTT effects. Excess ROS generation causes oxidative stress of tumor cells and directly cause DNA damage, which in turn activate downstream signaling pathways, and then induce death of tumor cells [46, 47]. Among of which, increasing evidence has shown that PI3K/AKT pathway could be regulated by cellular ROS and leads to mitochondria dysfunction [48, 49]. It has been well accepted that upon activation, AKT is phosphorylated by PI3K and therefore inactivates the pro-apoptotic protein Bax and protects cells from apoptosis. In addition, phosphorylated AKT is also able to stabilize the MDM2/p53 complex, which regulates cell survival [50]. In this context, the role of such pathway in CoFe_2_O_4_-QDs induced ROS secretion was investigated. As expected, we found that excessive ROS caused by CoFe_2_O_4_-QDs significantly downregulated the expression of PI3K/AKT pathways and therefore cause tumor cell apoptosis via activating Bax but inactivating Bcl-2 protein. This finding was further confirmed by adding ROS inhibitor, which reversed the PI3K/AKT expression and decreased the production of ROS. Since PI3K/AKT pathway is known to regulate cell survival and death, especially in cancer cells, understanding such mechanisms of CoFe_2_O_4_-QDs in killing NSCLC would help us to develop more options for combinational therapies.

In summary, to develop novel photosensitizers for alternative tumor killing therapy, we successfully constructed CoFe_2_O_4_-QDs by using hydrothermal approach in a low-cost and easy manner in this study. The CoFe_2_O_4_-QDs have a wide NIR absorbance, good biocompatibility and photothermal conversion ability. In addition, compared to previously reported QDs, CoFe_2_O_4_-QDs exhibited synergistic PTT/PDT effect in killing NSCLC tumors, which representing a promising multifunctional agent in further phototherapies of NSCLC. Moreover, with the NIR irradiation, CoFe_2_O_4_-QDs could kill NSCLC mainly through inducing ROS generation via regulating Bcl-2/Bax expression through the upstream PI3K/AKT signaling pathway. As for in vivo tumor killing ability, CoFe_2_O_4_-QDs combined with NIR could eliminate the NSCLC tumor xenografts completely without obvious toxic effects. These findings prove that CoFe_2_O_4_-QDs owns promising applications to be developed as a novel NSCLC killing reagent.

## Conclusion

All in all, CoFe_2_O_4_-QDs we synthesized could exhibit superior PTT/PDT synergistic effects in suppressing NSCLC by inducing ROS generation through regulating PI3K/AKT pathway, which shed light to the mechanism research and applications of novel photosensitizers establishments.

### Supplementary Information

**Additional file 1: Supplementary Figure 1**. In vitro ROS detection. Related to Fig. 5A and B. DCF FACS analysis shows the ROS level in NCI-H460 and A549 with diffrent treatments. Representative data are shown. N = 3 C and D. The protein level of P-PI3K and P-Akt were determined by WB. Reperesentative images are shown.

**Additional file 2: Supplementary Figure 2**. Related Fig. 6. A The Ki-67 IHC staining of tumer xenografts in each group 1 day after treatment. Reperesentative images are shown. Scale bar, 50 nm. N = 3. B The quantification of Ki-67 positive staining from IHC images. At least 10 frames (40X) were quantified. The data are shown as ±*SD*. **P < 0.01. C Reperesentative H&E staining of kidney, liver, lung, heart and spleen in control group and combinational treatment groups after mice were sacrificed. N = 5. Sacle bar, 50 nm.

## Data Availability

The data that support the findings of this study are available from the corresponding author upon reasonable request.
