# Correction: CoFe_2_O_4_-Quantum Dots for Synergistic Photothermal/Photodynamic Therapy of Non-small-Cell Lung Cancer Via Triggering Apoptosis by Regulating PI3K/AKT Pathway

**DOI:** 10.1186/s11671-023-03877-7

**Published:** 2023-08-21

**Authors:** Jingfeng Liu, Xiaoying Shi, Rongjun Zhang, Miaomiao Zhang, Juan He, Jian Chen, Zheng Wang, Qingwen Wang

**Affiliations:** 1 Department of Rheumatism and Immunology, Peking University Shenzhen Hospital, Shenzhen Peking University-The Hong Kong University of Science and Technology Medical Center, Shenzhen, 518036 Guangdong China; 2 Shenzhen Key Laboratory of Immunity and Inflammatory Diseases, Peking University Shenzhen Hospital, Shenzhen, 518036 Guangdong China; 3 Cardiovascular Hospital, No. 1 Hospital of Xi’an City, Xi’an, 710002 China; 4 Cancer Research Institute, School of Basic Medical Sciences, Southern Medical University, Guangzhou, 510515 Guangdong China; 5 School of Advanced Materials and Nanotechnology, Xidian University, Xi’an, 710126 China

**Correction: Discover Nano (2021) 16:120** 10.1186/s11671-021-03580-5

The original article [1] unfortunately contained an error in the “Synthesis of CoFe_2_O_4_‑QDs” part of the “Material and Methods” section:

Due to a typographical error “Diethanol amine” was incorrently presented as “Dithranol amine”.

The original article has been corrected.
