# Supplementary figures and images for: CoFe2O4-Quantum Dots for Synergistic Photothermal/Photodynamic Therapy of Non-small-Cell Lung Cancer Via Triggering Apoptosis by Regulating PI3K/AKT Pathway
Source: Nanoscale Res Lett. 2021 Jul 28;16:120. doi: 10.1186/s11671-021-03580-5 (PMC8319264; doi:10.1186/s11671-021-03580-5)

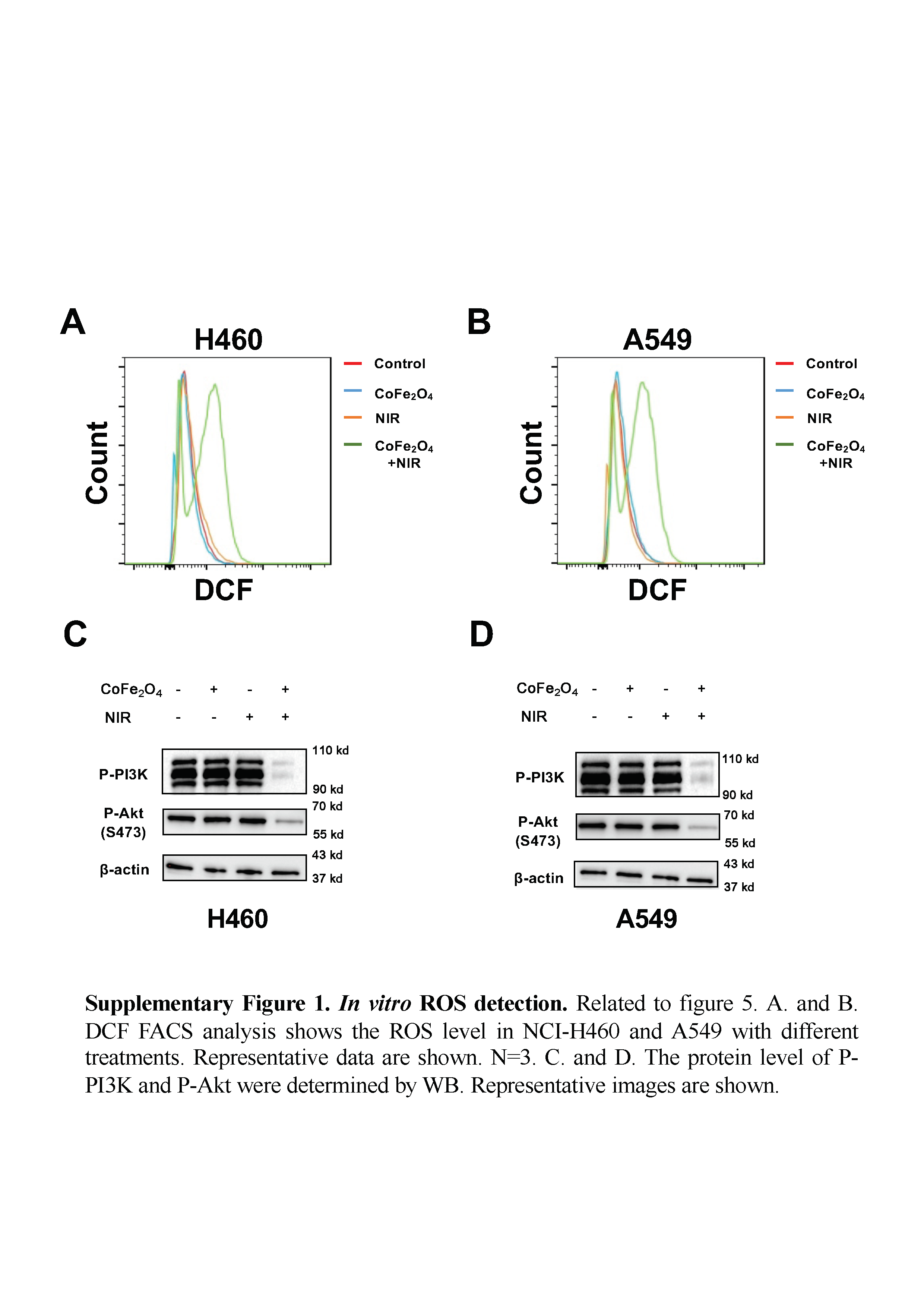

Supplement: Supplementary file 1 — Additional file 1: Supplementary Figure 1. In vitro ROS detection. Related to Fig. 5A and B. DCF FACS analysis shows the ROS level in NCI-H460 and A549 with diffrent treatments. Representative data are shown. N = 3 C and D. The protein level of P-PI3K and P-Akt were determined by WB. Reperesentative images are shown. [file 11671_2021_3580_MOESM1_ESM.tiff]

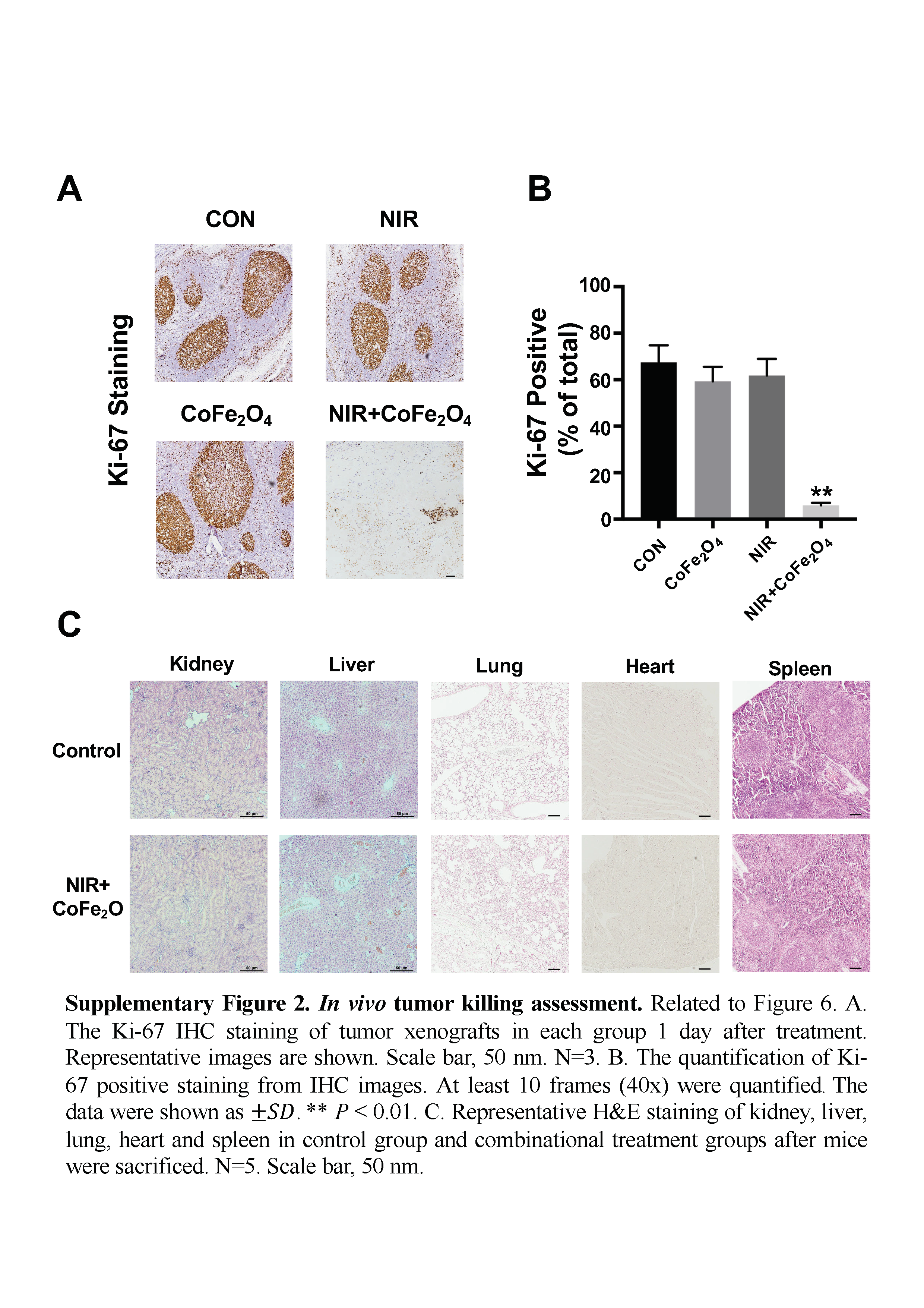

Supplement: Supplementary file 2 — Additional file 2: Supplementary Figure 2. Related Fig. 6. A The Ki-67 IHC staining of tumer xenografts in each group 1 day after treatment. Reperesentative images are shown. Scale bar, 50 nm. N = 3. B The quantification of Ki-67 positive staining from IHC images. At least 10 frames (40X) were quantified. The data are shown as ±SD. **P < 0.01. C Reperesentative H&E staining of kidney, liver, lung, heart and spleen in control group and combinational treatment groups after mice were sacrificed. N = 5. Sacle bar, 50 nm. [file 11671_2021_3580_MOESM2_ESM.tiff]
